# Letter to Editor: The smokeless tobacco habit and DNA damage: A systematic review and meta-analysis 

**DOI:** 10.4317/medoral.1122335667793

**Published:** 2019-12-28

**Authors:** Armen Nersesyan

**Affiliations:** 1 Department of Chemical Safety and Cancer Prevention, Institute of Cancer Research, Department of Internal Medicine I, Medical University of Vienna

De Geus *et al*. (1) carried out a systematic review and meta-analysis of the results obtained in the studies on DNA damage induced in oral (buccal) cells of smokeless tobacco users. The authors mentioned that “most of the recent studies have shown that the frequency of micronucleus (MN) is significantly higher in chewing tobacco [better to write “in tobacco chewers”] and snuff users than in non-users, but there is still a controversy in the literature”. The first part is completely true but the second one not at all. Indeed, the authors cited 3 papers which (in their opinion) reflect “controversy in the literature” (refs. 16 – 18). But two of them reported about positive results (increased MN levels in buccal cells of users) and only one (ref. 17) presented negative findings in buccal cells of 'maras' powder users but significant increase of MN frequencies (1.44-fold; *p* < 0.01) in exfoliated inner lip cells. It is important to mention that “maras” powder (one portion is 0.5 – 1.0 g) is applied to the lower lip mucosa and has no direct contact with buccal cells.

It is important to note that Proia *et al*. (2) published in 2006 a review paper concerning the same topic in which they showed clear increase of MN rates in exfoliated buccal cells of smokeless tobacco users (in total 23 studies). Some of these studies were also mentioned by De Geus *et al*. (1). In my opinion, the paper by Proia *et al*. (2) had to be mentioned by the authors.

It is absolutely not clear why the authors ignore current validated and standardized protocol to study MN in exfoliated buccal cells (3). In several their papers they state that scoring of 1000 exfoliated buccal cells stained with nonDNA specific stains is accepTable and the same results in MN assay can be achieved compared with results of studies carried out according to the protocol (4,5). Despite criticism (6,7) they continue to state the same.

In the validated buccal cells MN protocol is clearly written that 2000 differentiated cells should be scored for MN and 1000 cells for other nuclear anomalies, and the cells should be stained with DNA specific stain (3). Although the authors discussed advantages and disadvantages of staining techniques (pages e152-e153), they classified the results of the studies in very strange manner.

Only 4 investigations cited by De Geus *et al*. (1) correspond to requirements of current protocol for MN assay in buccal cells (refs. 1, 16, 23 and 24). There is also another paper (ref. 34) which was carried out according to the protocol (2000 Feulgen stained cells were evaluated) but this paper must be excluded from analysis because it concerns hookah (smoking but not smokeless tobacco use). Also, several papers should be excluded from the analysis in which less than 1000 cells were scored (i.e. refs 7, 13, 25 and 29) because the results of such studies are not reliable.

Extremely strange is assessment of publications quality and final rating of the studies carried out by the authors (Table 2). In this Table they collected all “strong” and “moderate” papers. It is not clear why the paper by Roberts (ref. 26) appeared in this Table indicated as “weak”; in this study 7600 cells were scored per person in exposed group and 1300 in the controls. As was mentioned above, the papers (refs. 7 and 13) should not be considered because of low number of scored cells, i.e. 20 (twenty!) and 300 respectively, but De Geus *et al*. classified these papers as “strong”! Also as “strong” is classified the paper concerning hookah smoking (ref. 34). All mentioned above articles should be excluded not only from Table but also from forest plot (Fig. 2). The paper by Livingston *et al*. (ref. 29) was classified as “moderate” although only 100 cells were evaluated in this study.

On page e146 the authors wrote that “Other chromosomal aberrations, such as pycnosis, binucleated cells, anucleated cells, are excellent biomarkers of exposure to the chromosome-damaging agents in tobacco”. Mentioned nuclear anomalies are not connected with chromosomal aberrations (genotoxicity), all they reflect cell death and are markers of cytotoxic effect (8-10).

On page e147 the authors wrote that “Other genotoxicity parameters were evaluated in some studies: nuclear buds, binucleated cells, anucleated cells, karyolysis, karyorrhexis, condensed chromatin, chromosomal aberrations and pycnosis”. On page e154 they called these nuclear anomalies as “genomic damage”. As mentioned above, nuclear buds, binucleates, anucleated cells, karyolysis, karyorrhexis, condensed chromatin and pyknosis are not connected with genotoxic events, they markers of cytotoxicity. The authors wrongly think that anucleated cells and karyolitic cells are two different categories.

Extremely strange is following statement of the authors (page e147, right column, paragraph 6): “Other genotoxicity parameters were evaluated in some studies: nuclear buds, binucleated cells, anucleated cells, karyolysis, karyorrhexis, condensed chromatin, chromosomal aberrations and pycnosis”. Why “chromosomal aberrations” are mentioned with so-called nuclear anomalies? 

Next shortcoming of the paper is wrong indication of magnification under which cells were evaluated. Motgi *et al*. (ref. 30), Sudha *et al*. (ref. 31), Pradeep *et al*. (ref. is absent in list of references) and Kohli *et al*. [ref. 22; by the way, in Tables the name is written with error - Kolhi] wrongly wrote that they used “x 40” or “x 100 magnification” for counting MN in the original papers. De Geus *et al*. (1) should know that “x 40” is not total magnification, it is just objective of the microscope. Magnification in this case is “x 400” because of “x 10” magnification of oculars and “x 40” of objective. No MN can be seen in exfoliated buccal cells under magnifications of “x 100” and, moreover, “x 40” 

Some literature sources are cited by the authors incorrectly. For example, a study concerning MN in female reproductive tract (ref. 9) is cited as a study the effects of environmental factors on genetic stability in human cells. As “an early marker of carcinogenesis” is cited a paper concerning MN level in buccal cells of petrol station attendants (ref. 10). Ref. 38 concerning advantage of Feulgen staining is also not precise since in this paper DNA specific stains “SYTOX green®” and propidium iodide were compared with Feulgen staining of lung carcinoma HK2 cells *in vitro*. It would be more correct in this case to cite the standardized and validated protocol for MN evaluation in exfoliated epithelial cells (3). The authors wrote that “It has been demonstrated in a recent systematic review (14) that smoking promotes a higher frequency of MN compared to non-smokers.” This statement of the authors is also not completely correct since the results of MN assay depend strongly on stain used in the study, i.e. high levels of MN in buccal cells of smokers were obtained mostly when DNA non-specific stains was used (for details see (7). It is also highly important in such studies to consider type of cigarettes consumed (tar and nicotine content), the number of cigarettes consumed per day and pack/years (11,12). The effects of duration of smoking on MN frequencies is questionable (11,12). In conclusion, there is no doubt that smokeless tobacco induces damage of genetic material and (due to high cytotoxic properties) also nuclear anomalies reflecting cytotoxicity. But writing the paper and presentation of results of other studies in chaotic manner for sure confuses the readers of the article by De Geus *et al*. (1).
